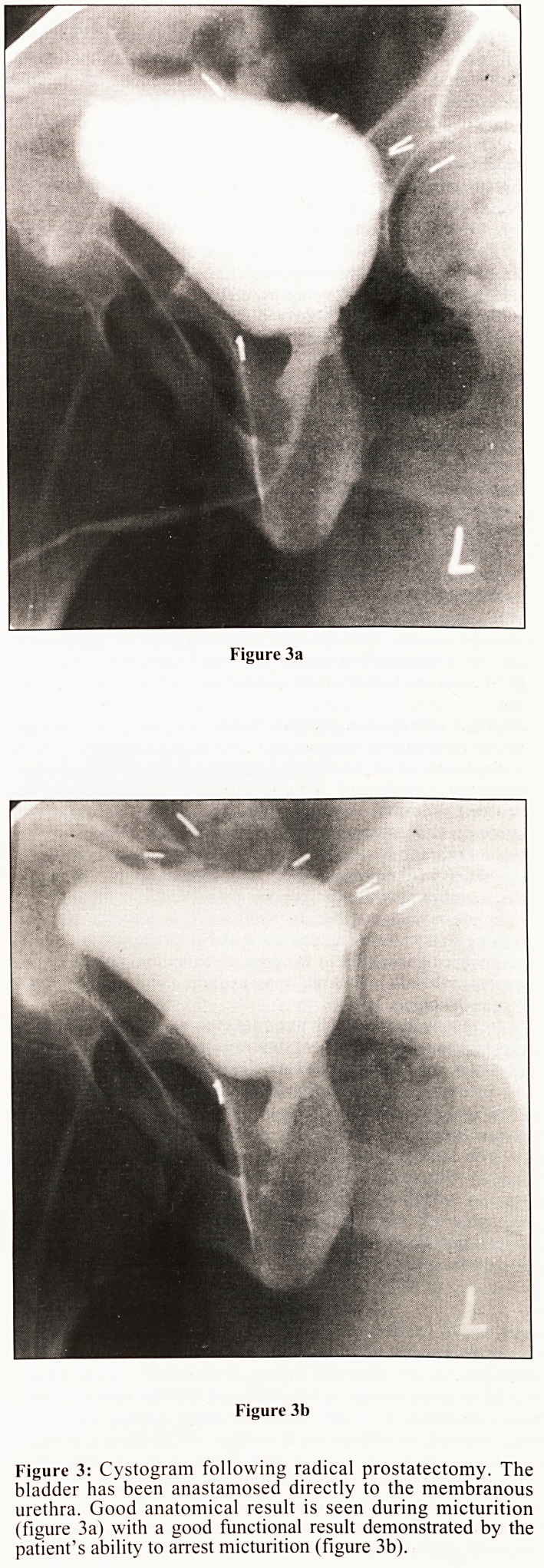# Current Trends in the Management of Localised Prostate Cancer

**Published:** 1992-12

**Authors:** G. N. A. Sibley, J. Kabala

**Affiliations:** Departments of Urology and Radiology Bristol Royal Infirmary; Departments of Urology and Radiology Bristol Royal Infirmary


### Current Trends in the Management of Localised Prostate Cancer

**G. N. A. Sibley and J. Kabala**
Departments of Urology and Radiology
Bristol Royal Infirmary

### INTRODUCTION

In the UK, carcinoma of the prostate is the third commonest cancer in men. Approximately 8,000 new patients present each year, and epidemiological data suggests that the incidence is steadily increasing. The majority of men affected are between 65 and 85 and the disease is rare before the age of 50.

Spread of the tumour occurs locally to the bladder trigone and posteriorly to the seminal vesicles, and in advanced cases the tumour may encircle the rectum. Lymphatic spread is initially to the pelvic lymph nodes, whilst haematogenous spread occurs chiefly to the axial skeleton (particularly the lumbar spine and pelvis) although other sites such as lung and liver are occasionally involved. Fifty per cent of patients have metastases at presentation (half of these are asymptomatic).

Treatment of prostate cancer in the UK has traditionally been relatively conservative. TUR of the prostate was performed for obstructive symptoms, and hormonal therapy (e.g. orchidectomy or the administration of oestrogens/anti-androgens) was given for metastatic disease. This policy was, in part, related to the relatively elderly age group predominantly affected by the disease. However, once metastases have developed the duration of response to hormonal therapy averages only 18 months, and progression of the disease is ultimately inevitable.

In recent years, there has been increasing enthusiasm for earlier detection of prostatic cancer when the disease is still confined to the prostate gland, in order that radical "curative" treatment could be instituted at this stage.

### CLINICAL DETECTION

Digital rectal examination (DRE) is an essential part of any full clinical examination and detection of an abnormal prostate on DRE (either a nodule or an irregularly hard prostate) remains the principal method of detection of prostatic carcinoma in the UK at the present time. This may occur in a patient complaining of typical "prostatic" voiding symptoms (e.g. hesitancy, poor stream, frequency), or the diagnosis may be suspected following DRE in a patient examined for non-urological symptoms (including symptoms from metastatic disease, e.g. bone pain). Histological confirmation of the diagnosis is, however, essential as 50% of palpable prostatic nodules are benign on biopsy.

Prostate specific antigen (PSA) is a serine protease specific for prostatic tissue that may be detected in small amounts in the peripheral blood. The serum level of PSA is elevated in 96% of patients with prostatic carcinoma and this has now replaced acid phosphatase as a marker of prostatic malignancy.The normal PSA is less than 4 ng/ml; elevation above this level should arouse suspicion of prostatic malignancy, although PSA levels may be elevated to a degree in benign prostatic enlargement also and hence histological confirmation is again required. Very high levels (>100 ng/ml) are indicative of metastatic prostatic carcinoma.

Screening of an asymptomatic population using a combination of DRE and PSA measurement has been suggested for the early detection of prostate cancer, and in the USA such screeing is common in men over the age of 40 undergoing routine annual health checks (prostate cancer is now the second most common cause of cancer-associated death in males in the USA). However, the value of a formal screening programme for prostate cancer in the UK remains arguable at the present time, and further evaluation of the potential benefits is required before advocating such a policy.

Although DRE and PSA measurement may raise the suspicion of an early prostate cancer, neither is a particularly sensitive indicator of the extent of the disease within the prostate nor of spread to adjacent structures (e.g. the seminal vesicles), both of which are crucial in determining which patients may be candidates for radical curative treatment. In addition, they are not helpful in the accurate assessment of lymph node involvement (which is associated with a poor prognosis that is unaltered by radical local treatment alone) and distant metastases. However, recent advances in imaging techniques have led to a steady improvement in the accuracy of staging in these patients.

### IMAGING TECHNIQUES FOR DIAGNOSIS AND STAGING

Transrectal ultrasound (TRUS) has become increasingly available in the last few years and has proved to be most effective in the diagnosis and local staging of carcinoma of the prostate.[1] Most carcinomas are of reduced echogenicity on ultrasound but other lesions, inflammatory and degenerative, can mimic this appearance. TRUS is significantly more sensitive than DRE for the detection of carcinoma (particularly the smaller, surgically more favourable lesions), but an appreciable number of false positives are encountered.[2] Transrectal biopsy using TRUS guidance (usually with an automated cutting needle through the biopsy channel on the dedicated ultrasound probe) is therefore generally performed at the same time (Fig. 1). Properly performed with adequate antibiotic cover, this is an efficient and safe procedure with a complication rate of less than 1% (generally minor urinary infection or haemorrhage).[3]

If radical prostatectomy is considered, then staging becomes critically important to exclude patients whose disease is no longer confined to the prostate and who would therefore be unlikely to benefit from the operation. DRE and CT scanning

**Figure 1:** Transrectal ultrasound scan through the right lobe of prostate. There is a large ill-defined echo-poor carcinoma lying peripherally (curved arrows). The line of white dots represents the projection of the biopsy guide. A biopsy with a cutting needle has been performed and air can be seen in the resultant track (straight arrows).

are insensitive for the detection of local spread, but TRUS has shown considerable usefulness in assessing capsular penetration and seminal vesicle invasion. Measurement of tumour size alone on TRUS may suggest inoperability, as tumours greater than 2.0 cm in diameter have a high likliehood of invasion through the capsule or into the seminal vesicles.[3]

More recently, considerable experience has been gained using magnetic resonance imaging (MRI) for staging prostatic carcinoma (Fig. 2). Although the accuracy of local staging may offer only a slight improvement on TRUS[4], MRI does allow an assessment of possible lymph node involvement. More accurate local staging may, however, become possible with the introduction of endorectal coils for MRI of the prostate, with initial reports suggesting an accuracy as high as 93%.[5]
Bone scintigraphy, using a $^{99m}$technetium-labelled isotope, remains the investigation of choice for excluding skeletal metastases in patients being considered for radical local treatment.

**Figure 2:** Transverse MRI scan of the prostate with good demonstration of the zonal anatomy. The peripheral zone is seen as a posteriorly lying crescent of higher signal. There is a small carcinoma present in the right lobe showing reduced signal (straight arrow). There is no evidence of extraglandular spread and in particular no apparent invasion of the adjacent neurovascular bundle (curved arrow).

### MANAGEMENT

Two groups of patients fall into the category of having locally confined prostatic carcinoma, where further treatment needs to be considered. In some patients, the disease is discovered histologically following TURP for obstructive symptoms thought to be due to benign prostatic enlargement (incidental carcinoma). The other group comprises patients diagnosed as having carcinoma at the outset, and in whom no previous treatment has been carried out.

### (a) Incidental carcinoma

Treatment depends on the extent and grade of the tumour in the prostatectomy specimen. If the tumour is well differentiated and involves less than 5% of the gland, the prognosis is excellent without treatment. Regular follow-up should be instituted to detect and treat the small proportion of patients who progress to more active disease.

In diffuse or multifocal disease, there is a greater risk of tumour progression and metastases. Further treatment should, therefore, be considered, especially in patients with less differentiated tumours. This usually involves external radiotherapy, although newer techniques such as implantation of radioactive iodine seeds[6] or laser therapy to the prostatic remnant[7] are currently under evaluation. Radical prostatectomy may be considered, but reconstruction may be difficult after TURP due to previous resection of the bladder neck.

**Figure 3a**

**Figure 3b**

**Figure 3:** Cystogram following radical prostatectomy. The bladder has been anastamosed directly to the membranous urethra. Good anatomical result is seen during micturition (figure 3a) with a good functional result demonstrated by the patient's ability to arrest micturition (figure 3b).

**(b) Locally confined carcinoma**

Patients with small, well differentiated tumours have a good prognosis and there is debate about the treatment that should be offered. Some centres favour surveillance, with deferred treatment for those in whom the disease progresses, and in one study of 122 patients with localised disease only 7% developed metastases at 5 years and there were only 2 cancer-related deaths in this time.[8] Other centres advocate radical treatment at the outset for all cases of locally confined prostatic cancer, a policy that seems difficult to justify in this specific group as it would be hard to better the results of surveillance alone.

In patients with diffuse involvement of the gland and in those with poorly differentiated tumours, the risks of local progression and of developing metastases are high (60% of patients with diffuse poorly differentiated tumour develop metastases within 3 years), and further treatment is therefore indicated at diagnosis.

**Radical radiotherapy** by external beam irradiation has been generally preferred in Britain, particularly in view of the elderly age group involved, and an overall 5-year survival of approximately 50% may be expected with this treatment.[9] The main advantage of radiotherapy is that it is non-invasive, but it may nevertheless be associated with significant side-effects, including proctitis, cystitis, lower limb or scrotal oedema, and impotence (in 40%).

Although some patients do have an excellent and long-term response to radiotherapy, post-irradiation biopsies from the prostate show an increasing incidence of positivity for tumour with time, with subsequent clinical relapse of the disease. This raises questions over the true radiosensitivity of the disease, but it is not possible at the present time to accurately determine those tumours that are fully radiosensitive from those that are not.

**Radical retropubic prostatectomy**, following preliminary pelvic lymphadenectomy to exclude nodal metastases, may be considered as an alternative to radiotherapy for younger patients (<70 years). This involves total removal of the prostate, seminal vesicles and distal vasa deferentia with subsequent anastomosis of the reconstructed bladder neck to the membranous urethra (Fig. 3). With careful anatomical dissection of the prostatic apex, the sphincteric mechanism of the membranous urethra can be preserved and incontinence rates are now low (2%). In addition, it is possible to retain potency in suitable cases by careful preservation of the neurovascular bundles to the corpora cavernosa (up to 70% of patients who are potent pre-operatively recover potency within 1 year of surgery).

In carefully selected patients, the results from radical prostatectomy are excellent, with up to 72% 10-year disease-free survival in patients where the tumour is confined within the prostate.[10] However, this figure drops dramatically to just 26% once the prostatic capsule has been breached and the seminal vesicles invaded.

True comparison of the relative merits of radiotherapy and radical surgery is difficult, due largely to the inaccuracies of staging in the non-surgically treated group. In particular, there has generally been a lack of data regarding the lymph node status in radiation-treated patients; this would tend to bias the results in favour of surgery, where node-positive patients are excluded. However, with the increasing accuracy of modern imaging techniques in detecting nodal metastases, a more accurate comparison of the two modalities should be possible in the future and should become the focus of clinical trials.

### SUMMARY

The incidence of prostate cancer in the UK is increasing, and the disease is being detected more often in younger patients (e.g. from routine PSA measurement during health-care screening). Left untreated, a significant proportion of patients will undergo progression of their disease locally and/or develop metastases.

Modern imaging techniques have greatly aided the assessment of early prostatic cancer, enabling both accurate assessment of the primary tumour and giving valuable information regarding lymph node metastases. PSA measurements are also extremely helpful, and this has replaced acid phosphatase as a marker for prostatic malignancy.

Controversy still remains, however, over the best form of management. Radical prostatectomy undoubtedly produces the best results in the literature, but the patients are highly selected (e.g. those with nodal metastases are excluded) and some patients with well differentiated tumours may have been over-treated, as they may have been expected to do well with surveillance alone. Full clinical trials are required in identically staged patients to assess the relative merits of surveillance, radiotherapy and surgery, and this should now be possible with recent advances in imaging techniques.

### REFERENCES

1. Clements R., Griffiths G.J., Peeling W.B. (1991). 'State of the art' transrectal ultrasound imaging in the assessment of prostatic disease. *Br.J.Radiol.*: **64**: 193-200.
2. Griffiths G.J., Clements R., Peeling W.B. (1989). The current status of transrectal ultrasonography in the diagnosis and management of prostatic cancer. *Clin.Radiol.*: **40**: 337-340.
3. Toi A. (1991). Transrectal prostate ultrasound and prostate cancer. *Diagnostic Oncology*. **1**: 111-119.
4. Rifkin M.D., Zerhouni E. A., Gastonis C. A., Quint L.E., Paushter D. M., Epstein J. I., Hamper U., Walsh P. C., McNeil B. J. (1990). Comparison of magnetic resonance imaging and ultrasonography in staging early prostate cancer. *New Eng.J.Med.*: **323**: 621-626.
5. Schnall M. D., Bezzi M., Pollack H. M., Kressel K. (1990). Magnetic resonance imaging of the prostate. *Magnetic Resonance Quarterly*: **6**: 1-16.
6. Holm H. H., Torp-Pedersen S., Myschetzky P. (1990). Transperineal seed-implantation guided by biplanar transrectal ultrasound. *Urology*: **36**: 249-252.
7. McNicholas T. A., Carter S.St. C., Wickham J. E. A., O'Donoghue E. P. N. (1988). YAG laser treatment of early carcinoma of the prostate. *Br.J.Urol.*: **61**: 239-243.
8. Adolfsson J., Carstensen J., Lowhagen T. (1992). Deferred treatment in clinically localised prostatic carcinoma. *Br.J.Urol.*: **69**: 183-187.
9. Read G., Pointon R. C. S. (1989). Retrospective study of radiotherapy in early carcinoma of the prosate. *Br.J.Urol.*: **63**: 191-195.
10. Hering F., Rist M., Roth J., Mihatsch M., Rutishauser G. (1990). Does microinvasion of the capsule and/or micrometastases in regional lymph nodes influence disease-free survival after radical prostatectomy? *Br.J.Urol.*: **66**: 177-181.

## Figures and Tables

**Figure 1 f1:**
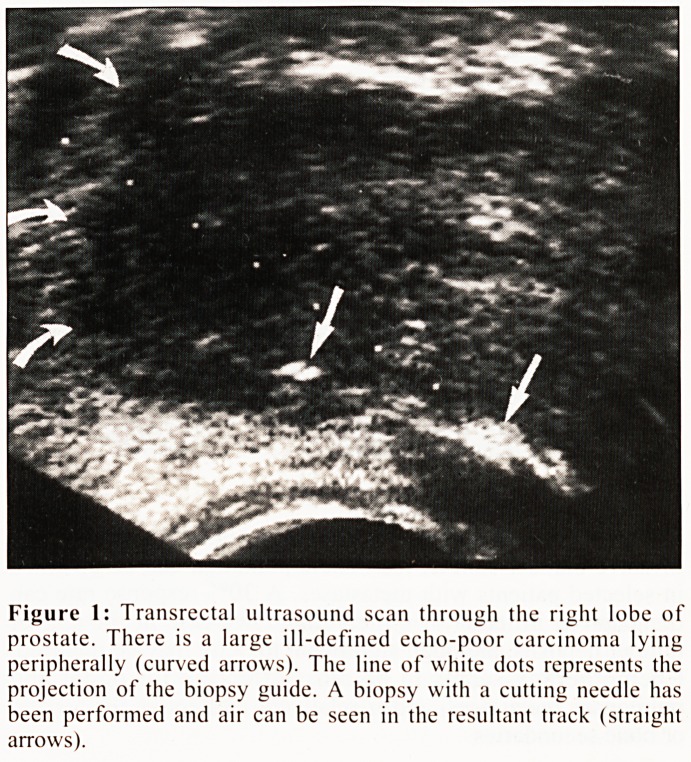


**Figure 2 f2:**
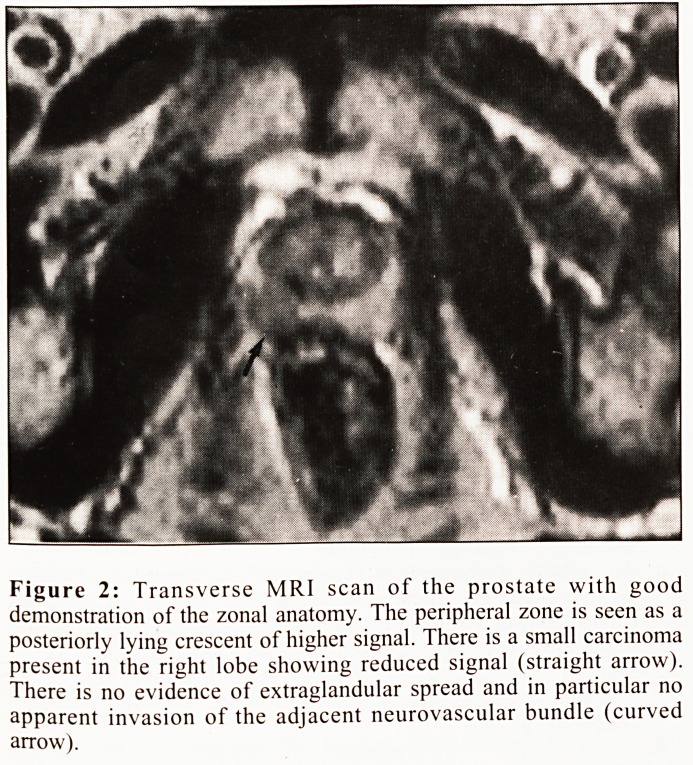


**Figure 3a Figure 3b f3:**